# Experimental design and data on the adsorption and photocatalytic properties of boron nitride/cadmium aluminate composite for Cr(VI) and cefoxitin sodium antibiotic

**DOI:** 10.1016/j.dib.2019.105051

**Published:** 2019-12-31

**Authors:** Rajeev Kumar, M.A. Barakat, Fathia A. Alseroury, Bandar A. Al-Mur, Md. Abu Taleb

**Affiliations:** a Department of Environmental Sciences, Faculty of Meteorology, Environment and Arid Land Agriculture, King Abdulaziz University, Jeddah, 21589, Saudi Arabia; b Central Metallurgical R & D Institute, Helwan, 11421, Cairo, Egypt; c Department of Physics, Faculty of Science, King Abdulaziz University, Jeddah, Saudi Arabia; d Department of Physics, Faculty of Science, University of Jeddah, Saudi Arabia

**Keywords:** BN/CdAl_2_O_4_ composite, Adsorption, Photocatalysis, Hexavalent chromium, Cefoxitin sodium

## Abstract

This article reports the experimental data on the adsorption and photocatalytic degradation-reduction properties of pure boron nitride (BN), cadmium aluminate (CdAl_2_O_4_) and boron nitride/cadmium aluminate (BN/CdAl_2_O_4_) composite for the hexavalent chromium (Cr(VI)) and cefoxitin sodium (CFT) in aqueous solution under the ultraviolet (UV) and visible light irradiation. This work evaluates the adsorption and photocatalytic efficiency of the 0.2g BN coupled with the CdAl_2_O_4_ in BN-0.2/CdAl_2_O_4_ composite for Cr(VI) and CFT. The experiments were performed by mixing the 0.025 material with 50 mL solution of known concentration (15 mg/L) at pH 3 for Cr(VI) and pH 7 for CFT. The obtained data can be valuable to select the proper light source (UV or visible) and pollutant to investigate the application of BN-0.2/CdAl_2_O_4_ composite. Moreover, presented data can help identify the equilibrium time for the adsorption process and to recognize the best process for the removal of the pollutants from wastewaters. A comparison of the obtained data with previously reported works has been conducted for the understanding of the adsorption and photocatalysis of Cr(VI) and CFT using various materials under the different experimental conditions.

**Table:** Specification Table

Subject	Environmental science, materials science
Specific subject area	Wastewater purification, adsorption, photocatalysis,
Type of data	Tables, Figures
How data were acquired	The concentration of the Cr(VI) before and after the adsorption and photocatalysis was analysed by HACH ChromaVer® 3 chromium reagent powder pillows. The concentration of the CFT before and after the adsorption and photocatalysis was analysed by UV–visible spectrophotometer. Diffuse reflectance spectra of the materials were recorded on Jasco-V-570 spectrophotometer, Japan.
Data format	Raw
Parameters for data collection	The experimental data were obtained to select the efficient light source for the photocatalytic properties measurement of the synthesized materials. The effect of time on Cr(VI) and CFT removal was studied in the dark (adsorption) and under UV and visible light irradiation (light intensity - 108 W) In addition, the selectively of the materials was evaluated for Cr(VI) and CFT degradation.
Description of data collection	The data related to adsorption and photocatalysis was collected by taking the fixed amount of the sample from the solution after a certain time interval. The adsorption was performed between 0 and 120 min. Thereafter, solutions were illuminated to UV or visible light up to 270 min.
Data source location	King Abdulaziz University, Jeddah, Saudi Arabia
Data accessibility	Raw data are provided with the article in supplementary file. Mendeley Data under Identification number: https://data.mendeley.com/submissions/ees/edit/nd4kffgwrb?submission_id=DIB_26802&token=ef96e766-ee8b-4068-8596-4f84855a6fbd
Related research article	Rajeev Kumar, M.A. Barakat, Bandar A. Al-Mur, Fathia A. Alseroury, Jamiu O. Enola. Photocatalytic degradation of cefoxitin sodium antibiotic using novel BN/CdAl_2_O_4_ composite. https://doi.org/10.1016/j.jclepro.2019.119076

**Table:** 

**Value of the Data** • Data obtained revealed that BN, CdAl_2_O_4_, and BN-0.2/CdAl_2_O_4_ composite are not very good adsorbents for the removal of Cr(VI) and CFT. • Data may be useful to design the new material with better adsorption and photocatalytic properties. • Data can be used to compare the different irradiation sources for photocatalytic degradation of organic and inorganic pollutants. • Data could be useful to select a proper light source for photocatalytic applications. • Data may be used to select the particular pollutant to investigate the photocatalytic properties of the synthesized materials.

## 1 Data description

**Fig. 1:**
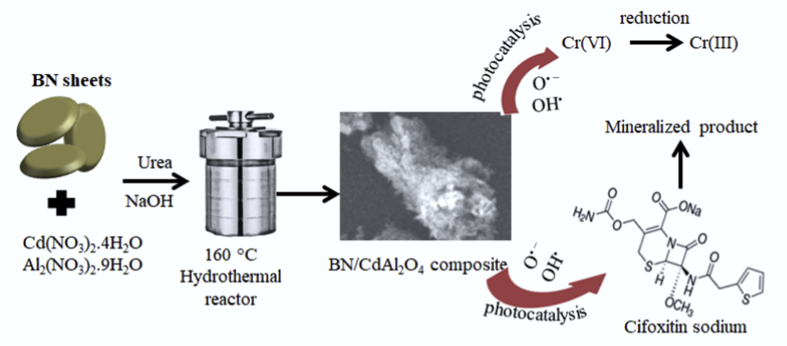
Schematic diagram for the synthesis of BN-0.2/CdAl_2_O_4_ composite and its photocatalytic application.

**Fig. 2:**
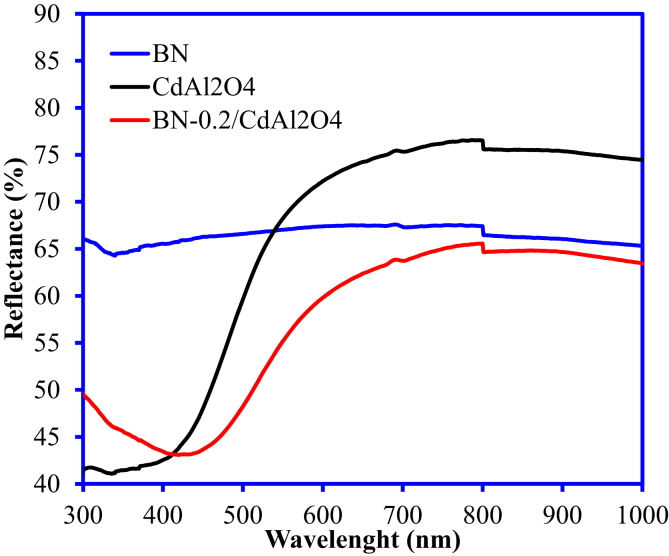
Diffuse reflectance spectra of BN, CdAl_2_O_4_ and BN-0.2/CdAl_2_O_4_ composite.

**Fig. 3:**
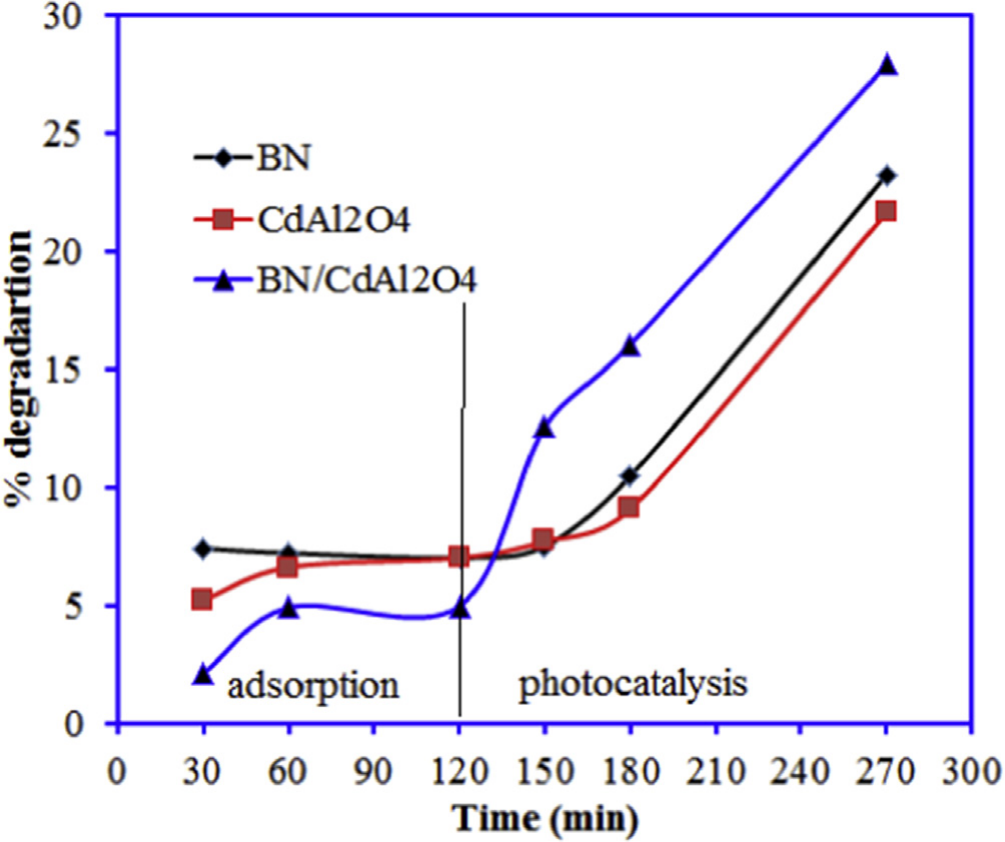
Adsorption and photocatalytic degradation of the CFT onto BN, CdAl_2_O_4_ and BN-0.2/CdAl_2_O_4_ composite under visible light irradiation (solution volume 50 mL, concentration 15 mg/L, pH 7, light intensity 108 W, catalyst mass 0.025 g).

**Fig. 4:**
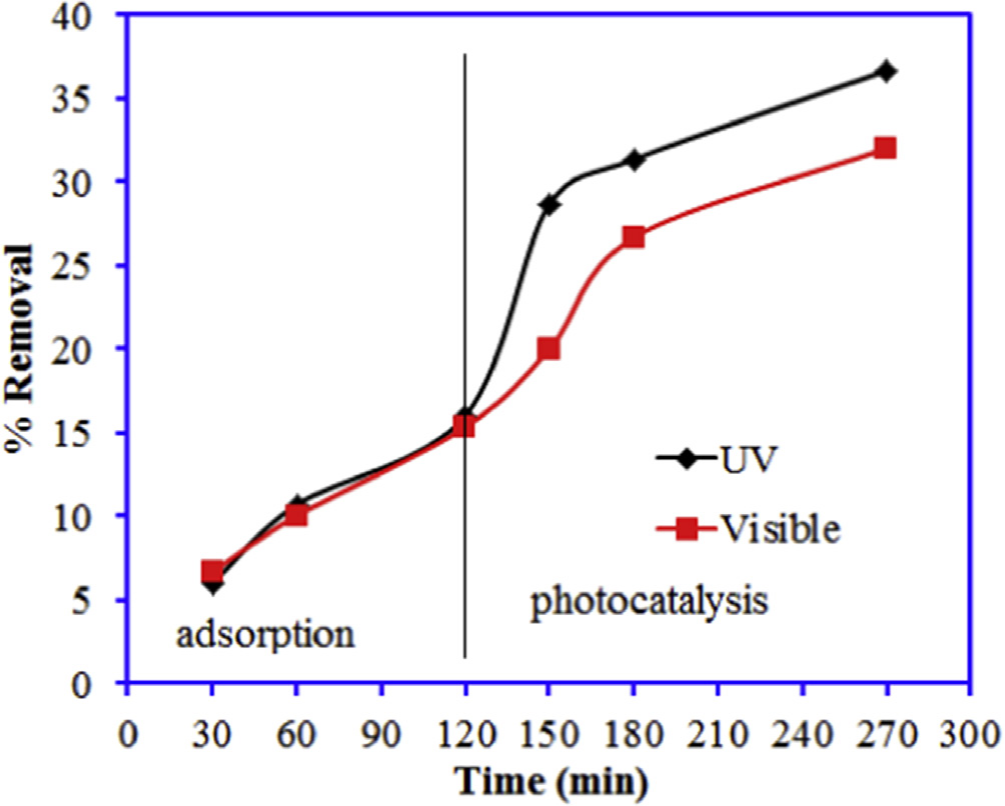
Adsorption and photocatalytic reduction of the Cr(VI) onto BN-0.2/CdAl_2_O_4_ composite under UV and visible light irradiation (solution volume 50 mL, concentration 15 mg/L, pH 3, light intensity 108 W, catalyst mass 0.025 g).

The incorporation of the BN with CdAl_2_O_4_ enhanced the photocatalytic of the synthesized BN-0.2/CdAl_2_O_4_ composite. However, BN-0.2/CdAl_2_O_4_ composite is not a very efficient catalyst in visible light. Data reported in this article is related to the article “Photocatalytic degradation of cefoxitin sodium antibiotic using novel BN/CdAl_2_O_4_ composite” [1]. This article reports why we selected the CFT and UV light source over the Cr(VI) and visible light for the study in Ref. [1]. A schematic diagram for the synthesis and photocatalytic properties of BN-0.2/CdAl_2_O_4_ composite is summarized in Fig. 1. The UV–visible diffuse reflectance spectrum of BN, CdAl_2_O_4_, and BN-0.2/CdAl_2_O_4_ composite is shown in Fig. 2. Table 1 shows the molecular formula and properties of the CFT. The photocatalytic properties of the BN, CdAl_2_O_4_, and BN-0.2/CdAl_2_O_4_ composite for CFT degradation under the visible light illumination is illustrated in Fig. 3. The photocatalytic efficiency of the BN, CdAl_2_O_4_, and a series of BN/CdAl_2_O_4_ composite prepared by varying the amount of BN from 0.1 to 0.4g, for the photocatalytic degradation of the CFT under 108 W UV light irradiating in reported in Ref. [1]. Herein, adsorption and photocatalytic efficiency of the BN-0.2/CdAl_2_O_4_ composite for Cr(VI) under UV and visible light irradiation is shown in Fig. 4. Raw data related to this articles mentioned in supplementary file.

**Table 1:** Properties of the cefoxitin sodium.

Molecular Formula	*λ*_max_	Molecular Formula	Molecular weight	Water solubility
	231 nm	C_16_H_16_N_3_NaO_7_S_2_	449.4 g/mol	soluble

A comparison of the experimental conditions, adsorption, and photocatalytic efficiency of the various materials reported previously for the removal of the CFT and Cr(VI) are summarized in Table 2 and Table 3.

**Table 2:** Comparison of photocatalytic properties of various materials used for the removal of Cr(VI) and CFT.

Catalyst	Pollutant	Light Source	Experimental Condition	% of removal	Ref.
BN/CdAl_2_O_4_	CFT	UV	pH-7, conc.-25 mg/L, time −240 min, catalyst mass-0.05g	84	[1]
Zn–Al-LDH	Cr(VI)	UV	pH-2, conc.-20 mg/L, time −150 min, catalyst mass-0.1g	60.49	[2]
LDH-TiO_2_	Cr(VI)	UV	pH-2, conc.-20 mg/L, time −150 min, catalyst mass-0.1g	95.53	[2]
rGO@LDO	Cr(VI)	Visible	pH-3, time −210 min. mass-0.1g	69.2	[3]
TiO_2_/AC-AEMP	Cr(VI)	UV	pH-2.5, conc.-40 mg/L, time −180 min, catalyst mass-0.25g	92.7	[4]
ZnO/PANI	Cr(VI)	UV	pH-4, conc.-20 mg/L, time-90 min, catalyst mass-0.5g	98	[5]
BN/CdAl_2_O_4_	Cr(VI)	UV Visible	pH-3, conc.-15 mg/L, volume-50 mL time-270 min. mass-0.025g	36.67 31.33	This work
BN/CdAl_2_O_4_	CFT	Visible	pH-7.23, conc.-14.5 mg/L, volume-50 mL, time-180 min, catalyst mass-0.025g	27.9	This work

**Table 3:** Comparison of adsorption properties of various materials used for the removal of Cr(VI) and CFT.

Adsorbent	Pollutant	Adsorption capacity (mg/g) or %	Experimental conditions	Ref.
Melanin	Cr(VI)	126.90	pH-3, adsorbent mass- 10 mg, time −3 h	[6]
Natural zeolite Cr(VI)-imprinted-poly (4-VP-coEGDMA)-ANZ	Cr(VI)	99.9	pH-2, volume −50 mL, adsorbent mass-0.0.08 g, time −120 min	[7]
g-C_3_N_4_/polyaniline nanofiber composite	Cr(VI)	187.58	pH-2, time −180 min, adsorbent mass- 0.015g, volume −25 mL	[8]
Chitosan-based hydrogel	Cr(VI)	93.03	pH-4.5, conc.-100 mg/L, volume-50 mL, adsorbent mass-0.1g	[9]
NBent-NTiO_2_-Chit	CFT	92.80%	pH-5, conc.-25 mg/L, adsorbent mass-0.15g	[10]
BN/CdAl_2_O_4_	Cr(VI)	2.3	pH-3, conc.-15 mg/L, volume-50 mL, time-120 min, adsorbent mass-0.025g	This work
BN/CdAl_2_O_4_	CFT	4.95%	pH-7.23, conc.-15 mg/L, volume-50 mL, time-180 min, adsorbent mass-0.025g	This work

## 2 Experimental design, materials, and methods

### 2.1 Materials

The model pollutant cefoxitin sodium (CFT) and potassium dichromate (salt for Cr(VI)) was supplied by Zhzhou Zhijun chemicals and BDH chemical, England. Otto chemical Ltd, India supplied boron nitride sheets. Cadmium nitrate and aluminum nitrate salts were received from BDH chemical Ltd. All the chemicals were used without further purifications. A freshly prepared solution was used for the adsorption and photocatalysis experiments by mixing the fixed amount of the salt in deionized water.

### 2.2 Synthesis and characterization

The synthesis of the CdAl_2_O_4_ and a series of BN/CdAl_2_O_4_ composites was performed in a 150 mL Teflon lined hydrothermal reactor at 160 °C. A detailed synthesis procedure and characterization of the BN, CdAl_2_O_4_, and BN/CdAl_2_O_4_ composites are reported elsewhere [1].

### 2.3 Adsorption and photocatalysis experiments

All the Cr(VI) and CFT adsorption and photocatalysis experiments were performed in 100 mL pyrex beaker under the dark and UV or visible light irradiation of 108 W intensity, respectively. Initially, 15 mg/L concentration solutions (500 mL) of Cr(VI) and CFT were prepared, and the pH was adjusted to 3 for Cr(VI) and pH 7 for CFT. The pH of the solution was adjusted using the 0.1 M HCl or 0.1 M NaOH solution. Thereafter, 0.025g of the material was added to the 50 mL solution of each pollutant for the adsorption studies in the dark. After 120 min in the dark, the solution was transferred into the LUZE CHEM photo-reactor for the photocatalysis experiment. Samples were collated after a fixed time interval to analyze pollutant concentration in the solution. The concentration of the Cr(VI) was analysed by the HACH-Dr6000 UV–visible spectrophotometer using HACH ChromaVer® 3 chromium reagent. The concentration of the CFT after the adsorption and photocatalysis was analyzed by UV–visible spectrophotometer at 235 nm. The adsorption and degradation efficiency of Cr(VI) and CFT was calculated by the following equation:

(1) % removal = (C_i_ – C_t_)/C_i_ x 100

where, C_i_ and C_t_, represent the initial and final concentration (mg/L) of the Cr(VI) or CFT at time t.
