# Supplementary material for: Experimental design and data on the adsorption and photocatalytic properties of boron nitride/cadmium aluminate composite for Cr(VI) and cefoxitin sodium antibiotic
Source: Data Brief. 2019 Dec 31;28:105051. doi: 10.1016/j.dib.2019.105051 (PMC7096680; doi:10.1016/j.dib.2019.105051)
Supplement: Multimedia component 2 [file mmc2.docx]

**Raw data**

| CFT adsorption and photocatalysis ( under visible light) | | | | |
| --- | --- | --- | --- | --- |
|  |  | % removal | % removal | % removal |
|  | Time (min) | BN | CdAl_2_O_4_ | BN-0.2/CdAl_2_O_4_ |
|  | 30 | 7.4 | 5.2 | 2.1 |
| Adsorption | 60 | 7.2 | 6.6 | 4.9 |
|  | 120 | 7 | 7 | 4.95 |
|  | 150 | 7.5 | 7.7 | 12.56 |
| Photocatalysis | 180 | 10.47 | 9.07 | 16.05 |
|  | 270 | 23.23 | 21.63 | 27.91 |

| Cr(VI) adsorption and photocatalytic reduction | | | |
| --- | --- | --- | --- |
|  |  | UV | Visible |
|  |  | % removal | % removal |
|  | Time (min) | BN-0.2/CdAl_2_O_4_ | BN-0.2/CdAl_2_O_4_ |
|  | 30 | 6 | 6.6 |
| Adsorption | 60 | 10.6 | 10 |
|  | 120 | 16 | 15.33 |
|  | 150 | 28.66 | 20 |
| Photocatalysis | 180 | 31.33 | 26.7 |
|  | 270 | 36.66 | 32 |
